# Somatic embryogenesis in Larix: the state of art and perspectives

**DOI:** 10.18699/VJ20.651

**Published:** 2020-10

**Authors:** V.N. Shmakov, Yu.M. Konstantinov

**Affiliations:** Siberian Institute of Plant Physiology and Biochemistry of Siberian Branch of the Russian Academy of Sciences, Irkutsk, Russia; Siberian Institute of Plant Physiology and Biochemistry of Siberian Branch of the Russian Academy of Sciences, Irkutsk, Russia Irkutsk State University, Irkutsk, Russia

**Keywords:** Larix, somatic embryogenesis, genetic control, Larix, соматический эмбриогенез, генетический контроль

## Abstract

Clonal propagation of conifers using somatic embryogenesis is essential for the selection of tree species, and for the implementation of afforestation and reforestation. In combination with cryopreservation, somatic embryogenesis creates the basis for the development of economically valuable lines of clones and elite genotypes. The industrial use of such genetically verified clone lines in forestry can significantly increase forest productivity compared to any conventional methods for improving tree crops that are available. Larch is considered as one of the main conifer candidates for large-scale reforestation, not only due to the vastness of its habitat, but also due to the unique quality of its wood, rapid growth and high ecological plasticity. However, the vast majority of larch species are characterized by uneven yields and extremely low seed quality. In this regard, obtaining planting material for reforestation from larch seeds on seed plantations is not advisable, but can be successfully implemented in afforestation programs using somatic embryogenesis technologies. Research on the somatic embryogenesis of larch has been conducted for over 30 years, which allowed considerable experience in this field to be accumulated. To date, the conditions for the initiation and maintenance of embryogenic cultures, as well as for the formation and development of somatic embryos have been determined. Significant progress has been made in the study of both the factors affecting these processes and the molecular mechanisms that underlie the various stages of embryogenesis. Nevertheless, despite the successes achieved, knowledge available today on the somatic embryogenesis of representatives of the genus Larix is still not enough to develop technologies for producing valuable plant-breeding material in vitro. This review analyzes the current state of research on the problem of somatic embryogenesis of representatives of the genus Larix. Particular attention is paid to the choice of explants for somatic embryogenesis, the composition of the media for cultivation, the dependence of the potential of somatic embryogenesis on the duration of cultivation, and the genetic control of somatic embryogenesis.

## Abstract (ru)

Клональное размножение хвойных с использованием соматического эмбриогенеза имеет суще- ственное значение для селекции древесных видов, реализации программ лесоразведения и лесовосстановле- ния. В сочетании с криоконсервацией соматический эмбриогенез создает основу для получения хозяйственно ценных линий клонов и элитных генотипов. Использование в промышленных масштабах в лесном хозяйстве та- ких генетически проверенных линий клонов может значительно увеличить продуктивность лесов по сравнению с любыми доступными традиционными методами улучшения древесных культур. Лиственница считается одним из основных кандидатов для широкомасштабного лесовосстановления не только за счет обширности занима- емых ареалов, но и благодаря уникальному качеству ее древесины, быстрому росту и высокой экологической пластичности. Однако большинство видов лиственницы характеризуется неравномерностью урожаев и чрез- вычайно низким качеством семян. В связи с этим получение посадочного материала для лесовосстановления из семян лиственниц на семенных плантациях нецелесообразно, но может быть успешно реализовано в програм- мах по лесоразведению с применением технологий соматического эмбриогенеза. Исследования по соматиче- скому эмбриогенезу лиственницы проводятся уже более тридцати лет, что позволило накопить значительный опыт в данной области. К настоящему времени изучены условия инициации и поддержания эмбриогенных культур, формирования и развития соматических зародышей. Достигнут значительный прогресс в изучении как факторов, влияющих на эти процессы, так и молекулярных механизмов, лежащих в основе различных этапов эмбриогенеза. Однако имеющихся на сегодняшний день знаний о соматическом эмбриогенезе пред- ставителей рода Larix все еще недостаточно для разработки технологий получения селекционно-ценного растительного материала in vitro. В обзоре проведен анализ современного состояния исследований по про- блеме соматического эмбриогенеза представителей рода Larix. Особое внимание уделено вопросам выбора эксплантов для соматического эмбриогенеза, составу сред для культивирования, зависимости потенциала со- матического эмбриогенеза от продолжительности культивирования, генетическому контролю соматического эмбриогенеза.

## Introduction

Representatives of the genus Larix are widespread in the cooltemperate and cold (subarctic and subalpine) regions of the planet (Gowere, Richards, 1990; Kim, 2015). The genus Larix includes 10 to 25 species, native to the northern hemisphere of three continents: North America, Europe and Asia (Dylis, 1981; Koropachinsky, Milyutin, 2013; Pâques et al., 2013). In the forest fund of our country, larch forests are uppermost both in area (about 37 %, 264 million hectares) and in wood stock (31 %, 25.2 billion m^3^); these markers surpass other species significantly (Efremov, Milyutin, 2010; Rysin, 2010). The issue of the exact number of larch species is to some extent controversial due to the ease of crossing in vivo and the production of hybrids, which, in turn, continue to hybridize (Wei, Wang, 2003; Koropachinsky, Milyutin, 2013).

Larch is considered one of the main candidates for extensive reforestation not only due to the vastness of its habitat, but due to the unique quality of its wood, rapid growth, and high ecological plasticity, too (Gowere, Richards, 1990; Bailian, Wyckoff, 1994). Larch is the only widespread polytypic deciduous conifer genus. It is a unique characteristic of this woody plant. Some way due to this quality, many larch trees can withstand extreme winter temperatures and low humidity levels (Bonga et al., 1995). The practical use of representatives of the genus Larix for reforestation is very effortful due to the low production and quality of seeds (Lelu et al., 1994a; Zhang Y. et al., 2012; Tretiakova et al., 2015). Therefore, it is not practical to obtain planting stock for reforestation from larch seeds in seed orchards. This problem can be solved using the methods of clonal propagation of embryos or seedlings derived from a limited number of seeds (Munoz-Concha, 2017). With Larix this has been done by grafting and rooting of stems taken from young seedlings and grown in a greenhouse. An alternative to this method is the use of the somatic embryo cultivation system, which makes it possible to derive an unlimited number of seedlings with the same genetic composition since they are derived from the same seed (Attree, Fowke, 1993; Isah, 2016).

Somatic embryogenesis can be much more efficient than traditional grafting. Cell lines produced by somatic embryos can be maintained in a juvenile state during indefinitely long time by their cryopreservation. Cryopreservation allows much longer field testings of cloned lines, while some of these lines are maintained in a physiological juvenile state until field testings show which of these lines are most preferable for mass propagation. That makes selection within families possible, which is not applicable to rooting by cuttings (Park, Bonga, 1992; Bonga, 2016). Moreover, the technology of using somatic embryogenesis can accelerate traditional reforestation programs by reducing the time required to obtain genetically improved trees (Kim, 2015). For conifers, unfortunately, the extensive use of somatic embryogenesis for practical propagation is often limited to only a few selected genotypes, and, in general, this process is still effortful and expensive. It is necessary to solve many problems for its universal use (Bonga, 2016; Klimaszewska et al., 2016).

This review is devoted to the analysis of the current status of research on the problem of somatic embryogenesis in representatives of the genus Larix. Hopefully, the acceleration of scientific progress in this area (due to the use of genomics, transcriptomics, proteomics, and metabolomics) will allow the development of new, more effective protocols of somatic embryogenesis for implementation in breeding, afforestation, and reforestation.

## Choice of the explant type for somatic embryogenesis in larch

For the first time, the method of somatic embryogenesis in Larix was successfully applied in 1985 for Larix decidua (Nagmani, Bonga, 1985). Since that time, great progress has been made in this field for most larch species and its hybrids. Table 1 shows Larix species in which somatic embryogenesis was obtained and the first references. As noted in numerous studies, the formation of embryogenic cultures depends on the type and stage of development of the explant.

**Table 1.**
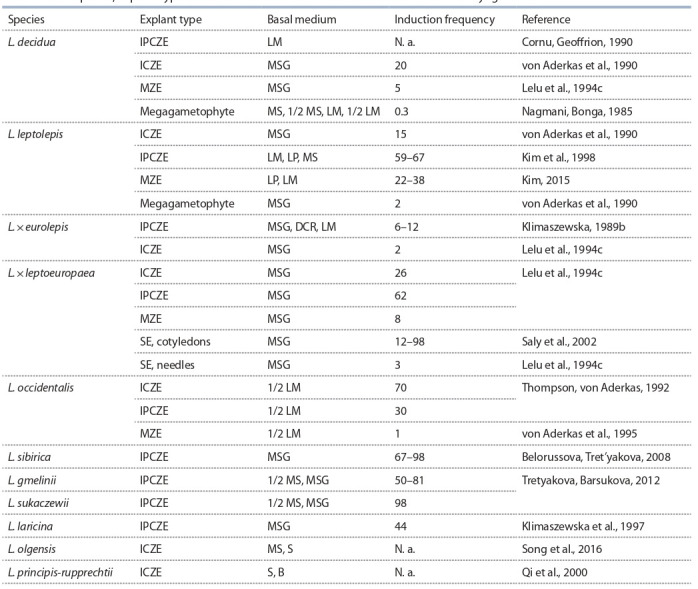
Larix species, explant type and basal cultural medium used to initiate somatic embryogenesis Notе. Explant type: MZE – mature zygotic embryo, IPCZE – immature precotyledonary zygotic embryo, ICZE – immature cotyledonary zygotic embryo, SE – somatic embryo.

Initial work was mainly carried out using megagametophytes L. decidua, L. leptolepis (=L. kaempferi) and their reciprocal hybrids L. × eurolepis and L. × leptoeuropaea as explants (von Aderkas et al., 1987, 1990; von Aderkas, Bonga, 1988; Rohr et al., 1989). Using the megagametophytes made it possible to obtain haploid embryogenic cultures and, on their basis, somatic embryos. The plants formed from the latter were most often mixoploids with a predominance of diploid cells (von Aderkas, Anderson, 1993; von Aderkas, Bonga, 1993; von Aderkas et al., 2002). In the same years, successful attempts were made to initiate somatic embryos from protoplasts of L. decidua and a hybrid of L. × eurolepis (L. decidua × L. leptolepis) (Klimaszewska, 1989a; von Aderkas, 1992; Korlach, Zoglauer, 1995; Pattanavibool et al., 1998). However, according to the research carried out in subsequent years, somatic embryogenesis is initiated most efficiently in immature zygotic embryos. This fact was determined both for species of the genus Larix and for other representatives of conifers (Chen et al., 2010; Bonga, 2016; Sarmast, 2018; Shuklina, Tret’yakova, 2019). In this case, pre-cotyledonary and cotyledonary zygotic embryos are most often used (Lelu et al., 1994a; Ogita et al., 1999a; Lu et al., 2005; Lelu-Walter, Pâques, 2009), although positive results were obtained when using zygotic embryos of earlier or later stages of development (Wang et al., 2007; Belorussova, Tret’yakova, 2008; Wang, Yang, 2010). There is little progress in initiating somatic embryogenesis in mature embryos. Thus, for the L. × leptoeuropaea hybrid (L. leptolepis × L. decidua), needles of somatic seedlings produced embryonic masses with a lower frequency (3 %) than mature somatic embryos of the same genotype (83 %) (Lelu et al., 1994c). Much more data on the successful initiation of somatic embryogenesis in mature embryos have been obtained to date for representatives of other taxonomic groups of conifers. Thus, embryogenic cultures were derived from mature zygotic embryos of Pinus geradiana, P. kesya, P. koraiensis, Abies alba, A. nordmanniana, A. balsamea, A. fraseri, Picea abies, P. glauca, P. morrisonicola, P. likiangensis, P. omorika, and others (Yeung, Thorpe, 2005; Vooková, Kormuťák, 2007; Chen et al., 2010; Shuklina, Tret’yakova, 2019).

A significant disadvantage of using juvenile material (zygotic embryos) as explants for clonal propagation, including using somatic embryogenesis, is the impossibility of using the material from plants whose genetic potential has already appeared phenotypically (adult trees with strictly defined characteristics) (Bonga, 2017). Therefore, the use of vegetative parts of plants (segments of shoots and mature needles) as a primary explant is of greatest interest. Thus, there are a number of studies indicating the possibility of obtaining somatic embryogenesis from plant material from adult coniferous plants: primordial meristems of 2- and 10-year-old somatic plants Picea glauca (Klimaszewska et al., 2011; Klimaszewska, Rutledge, 2016), needles of 2-month- to 3-year-old somatic plants Picea abies (Harvengt et al., 2001), vegetative apexes of shoots, adventive buds and needles of adult trees Pinus kestiya, P. patula, P. roxburghii, P. sylvestris, P. wallichiana, P. pinea, P. radiata, P. pinaster (Trontin et al., 2016a; Shuklina, Tret’yakova, 2019).

Difficulties in using plant material from adult trees are associated with the fact that significant physiological, biochemical, genetic and other changes in plant tissues occur at the vegetative and reproductive stages of development of adult trees. That significantly reduces the likelihood and frequency of embryogenesis from explants of adult trees (Klimaszewska et al., 2011). While the explant maturity increases, the genetic program for the induction of embryogenic tissue is gradually repressed; after the formation of the apical meristem, the potential for obtaining embryogenic tissue is completely disrupted (Bonga et al., 2010).

Other negative factors include the following: due to the presence of many compounds formed and released into nutrient media by plants, cell division and growth are inhibited, breakages of embryogenic response and normal expression of genes involved in the induction of somatic embryogenesis are observed (Isah, 2016; Sarmast, 2018). One of the important reasons for the above breakages is a change in the genomic DNA methylation pattern (von Aderkas, Bonga, 2000). To overcome the inability of the material of adult plants to form an embryogenic culture, the procedure of tissue rejuvenation is used in some cases. For this purpose, explants are placed in various kinds of stressful conditions: fasting, cold treatment at low positive temperatures, or using heavy metals as part of culture media (Bonga, 1996, 1997; Wendling et al., 2014). Rejuvenation is stimulated by a change in culture medium pH, use of enzymes of cell wall degradation, and decrease in the level of endogenous antioxidants (glutathione, ascorbic acid, vitamin E) by changing the content of exogenous auxins (von Arnold, 1987; Earnshaw, Johnson, 1987; Mo et al., 1996). The effect of auxin has been shown to increase the level of DNA methylation, which, in turn, leads to stimulation of cell division and their dedifferentiation; thus, it can stimulate the initiation of somatic embryogenesis (von Aderkas, Bonga, 2000).

In addition, tissues of adult coniferous trees contain many surface microorganisms reducing the tissue’s ability to regenerate (Tretiakova et al., 2014). Unfortunately, to date, no effective methods have been developed to combat such bacterial contamination. Only a few procedures are known to reduce but not completely eliminate such contamination in plant tissue culture systems (Sarmast, 2018).

Despite decades of research efforts (Chalupa, 1989; Bonga, Pond, 1991; Bonga, 1996, 2004; Ewald, 1998), somatic embryogenesis from adult larch trees has not been derived down to recent times; it remains an urgent task for future research (von Aderkas, Bonga, 2000; Klimaszewska et al., 2016).

## Effect of the parent plant genotype on the somatic embryogenesis in larch

The genotype of parental trees is much more likely the main factor (in addition to the explant type) determining whether somatic embryos will form (Tret’yakova, Barsukova, 2010; Bonga et al., 2010). In such a case, the initiation of somatic embryogenesis in many conifers is influenced by additive genetic variability, which provides an opportunity for selection to enhance the initiation pattern of somatic embryogenesis (Park, 2002). Somatic embryogenesis can be obtained only from certain genotypes; that significantly complicates the use of this technology for practical extensive propagation of conifers.

Experiments to study the genetic control of the initiation of somatic embryogenesis were carried out on representatives of several conifer genera, including the genus Larix (Klimaszewska et al., 2016). In Tretyakova et al. (Tretyakova et al., 2015), 200 L. sibirica trees were used. However, only one genotype initiated a stably maintained embryogenic culture. Further, the analysis of the results obtained indicates a stronger maternal than paternal effect on the culture initiation (Tretyakova et al., 2015; Klimaszewska et al., 2016). The maternal effect at the initiation stage can be explained by both the genotype and the stage of development or physiological state of the maternal tree, as well as inherited maternal alleles of the zygotic embryo (Niskanen et al., 2004).

## Composition of culture media for embryogenic masses and somatic embryos of larch

The growth and development of the embryogenic culture of conifers, including representatives of the genus Larix, is strongly influenced by the nutrient medium composition. The choice of salts (micro- and macroelements) and organic components, as well as correction of their balance and concentrations play an important role both in the induction of callusogenesis and in the further maintenance of the resulting culture in vitro (Pâques et al., 2013). Moreover, the choice of the nutrient media composition often depends on the plant species and the type of material used as an explant (Isah, 2016).

The following basal media are used in studies on the somatic embryogenesis in larch, depending on the tasks to be solved and the initial plant material (see Table 1): LM (full or half strength (1/2 LM)) (Litvay et al., 1985), MS (full or half strength (1/2 MS)) (Murashige, Skoog, 1962), MSG (Becwar et al., 1990), LP (Quoirin, Lepoivre, 1977), S (Ewald et al., 1995), B (Ewald et al., 1997), AI (Tret’yakova et al., 2012), DCR (Gupta, Durzan, 1985), WPM (Lloyd, McCown, 1980). During all the stages of cultivation, the basal medium is supplemented with such organic compounds as: L-glutamine (0.05–1.5 g/L); myo-inositol (0.1–1.0 g/L); casein hydrolyzate (0.5–1.0 g/L); ascorbic acid (0.4 g/L) (Cornu, Geoffrion, 1990; von Aderkas et al., 1990; Lelu et al., 1994c; Klimaszewska et al., 1997; Kim, 2015; Tretyakova et al., 2015).

Phytohormones are key components of the nutrient medium that control the entire process of somatic embryogenesis (von Aderkas et al., 2001; Vondráková et al., 2016). Moreover, their composition and ratios depend on the development stage of somatic embryos. During the induction of embryogenic masses, the presence of endogenous auxins in combination with cytokinins is necessarily in the medium. The exception is species Abies, in which only cytokinins are most often required for the induction of embryogenesis (Pullman, Frampton, 2018). The use of 2,4-dichlorophenoxyacetic acid (1.0–2.0 mg/L) in conjunction with 6-benzylaminopurine (0.5–1.0 mg/L) is shown in the overwhelming majority of studies on embryogenesis in representatives of the genus Larix (Klimaszewska, 1989a, b; Korlach, Zoglaue, 1995; Lelu-Walter, Pâques, 2009; Tretyakova et al., 2019). In a number of studies, naphthylacetic acid, picloram, or 4-chlorophenoxyacetic acid at 1.0 mg/L (Qi et al., 2004; Kim, 2015) and kinetin (0.1–5.0 mg/L) as a representative of cytokinins (Cornu, Geoffrion, 1990; Qi et al., 2000; Song et al., 2016) are mentioned as auxins. Indoleacetic acid, as shown for L. leptolepis, plays an important role in controlling the germination of somatic embryos (Li Z. et al., 2017a, b).

At the maturation of somatic embryos, abscisic acid becomes the most important component of the nutrient medium (Lelu et al., 1994b, 1995). The optimal content of this phytohormone (0.01–32.0 mg/L) and the cultivation time of somatic embryos in its presence (1–4 weeks) vary significantly in different larch species (Label, Lelu, 1994, 2000; von Aderkas et al., 1995, 2002, 2015; Gutmann et al., 1996; Klimaszewska et al., 1997; Ogita et al., 1999b; Kim, Moon, 2007; Tret’yakova et al., 2012; Song et al., 2018). Sometimes, indolebutyric acid at 1.0 mg/L (Tret’yakova et al., 2012), 5.0 mg/L of auxin transport inhibitor 2-(p-chlorophenoxy)-2-methylpropionic acid (PCIB), 5.0 mg/L phloroglucinol (auxin synergist) (Kim, Moon, 2009) or silver nitrate (2.0–5.5 mg/L) (Saly et al., 2002; Song et al., 2018) are used together with abscisic acid to improve the process of maturation of somatic embryos. Improving the quality of somatic embryos, their germination and the formation of full-fledged plants is achieved by combining abscisic acid with activated carbon (0.5–10 g/L), which is introduced into the nutrient medium during the pre-maturation of somatic embryos (Harry et al., 1991; Qi et al., 2004; Umehara et al., 2004; Klimaszewska et al., 2016; Tretyakova et al., 2016). Considering that maturing somatic embryos should be exposed to water stress, similar to developing zygotic embryos in vivo, substances such as polyethylene glycol 3000–4000 at 4–10 %, sucrose at an increased concentration (3–8 %) or maltose (3 %), and gelling agents gelright or phytagel (0.3–0.4 %) are introduced into the nutrient medium in order to reduce the available water (Klimaszewska et al., 1997; Ma et al., 1998; Qi et al., 2004; Lu et al., 2005; Teyssier et al., 2011; Tret’yakova, Barsukova, 2012; Tret’yakova et al., 2012; Song et al., 2018). At the initial stages of the induction of the embryogenic culture development, the concentration of sucrose used is 1–3 % (von Aderkas et al., 1987; Lelu et al., 1994c; Kim, 2015); agar at 0.7 % is most often used as a gelling agent (Klimaszewska, 1989b; von Aderkas et al., 1990; Belorussova, Tret’yakova, 2008).

In addition to studying the positive effect of certain compounds making up the culture media on various stages of somatic embryogenesis, we also studied substances whose presence in the medium negatively affects the culture in vitro. For L. × leptoeuropaea, it was shown that the atmosphere enrichment with ethylene or the addition of 2-chloroethylphosphonic acid (5.0 and 10.0 mg/L) or 1.0–10.0 mg/L of 1-aminocyclopropane-1-carboxylate to the culture medium greatly reduced the induction of secondary somatic embryogenesis (Saly et al., 2002). In L. leptolepis, vanillyl benzyl ether and 4-[(phenylmethoxy)methyl] phenol inhibited the early development of somatic embryos, namely, the differentiation of suspensors (Umehara et al., 2005, 2007). These substances were shown to be present in sufficient for inhibition amounts in a high cell density suspension culture, while they were at significantly lower, non-deleterious concentrations in a low cell density culture (Umehara et al., 2004).

To date, extensive experience has been accumulated in the field under study. However, due to the still low efficiency of somatic embryogenesis of representatives of the genus Larix, work on optimizing the nutrient medium composition, including specific sugars, vitamins, organic acids and modifiers of redox potential, etc. continues.

## Dependence of somatic embryogenesis potential of larch trees on the culture age in vitro

In works on the induction and maintenance of an embryogenic culture, a serious attention is paid to the efficiency of obtaining somatic embryos in culture in vitro during long periods of time. The issue is of both fundamental and applied importance for reforestation programs requiring long-term regenerated trees testing from seperate cell lines to their extensive use. Consequently, tissue culture lines should be maintained in a functionally unchanged form until the elite characteristics of the regenerants derived from them are experimentally confirmed (Charest, Klimaszewska, 1995). Although, the age of the embryogenic culture, i. e., the number of subcultures, can undermine their ability to regenerate full-fledged somatic embryos (Pâques et al., 2013). That may be primarily connected with an increase in the rate and accumulation of a large number of mutations and general genetic instability of cultures kept in vitro for a long term as a result of somaclonal variability (Krutovsky et al., 2014; Klimaszewska et al., 2016).

Somaclonal variability can appear on morphological, cytological (number and structure of chromosomes), biochemical (metabolic disorders) and molecular genetic (nucleus and organelle genomes) levels (Cyr, Klimaszewska, 2002). Partially differentiated cultures, such as in vitro embryonic masses, were found to show less variability than true callus-type cultures (Cyr, 1999). Moreover, embryogenic coniferous cultures are considered genetically more stable as opposed to angiosperms (Isabel et al., 1996). A number of studies assessing the level of somaclonal variability in embryogenic cultures of conifers showed the absence of any somaclonal changes in embryogenic tissues and in somatic embryos of Picea abies, Picea glauca × P. engelmannii, Pinus pinea, Picea mariana (Heinze, Schmidt, 1995; Isabel et al., 1996; Cuesta et al., 2008; Krutovsky et al., 2014). In general, it is seen that embryogenic cultures of representatives of the genus Larix have a relatively high stability (Klimaszewska et al., 2016). Thus, in L. × eurolepis, the embryogenic line was stable after 4 years of subculturing (Pâques et al., 2013), and in L. leptolepis it was stable for 9 years (Wang et al., 2007; Lelu-Walter, Pâques, 2009). In the latter case, the embryogenic cultures became non-embryogenic over the course of time (Li W. et al., 2013). The embryogenicity of L. decidua cultures derived from haploid material (megagametophytes) was not lost for 9 years (Pattanavibool et al., 1995). In such case, almost all lines doubled (2n = 24) their number of chromosomes during the observation period, but both haploid and dihaploid lines remained embryogenic. Further studies (17-year-old culture) showed that none of the lines retained constant embryogenicity during the course of the entire cultivation period (von Aderkas et al., 2003). In several lines, the embryogenic potential was completely lost, while in others the loss was temporary since there was an embryogenesis periodic restoration. The proliferative activity of 15 embryogenic cell lines of L. sibirica persisted for 2–8 years (Pak et al., 2016; Tretyakova, Pak, 2018). The chromosome ploidy of the cells of these lines did not change until two years of cultivation (Tretyakova et al., 2017). Further, a scatter of chromosome numbers from 24 to 30 and a large number of mitosis and micronuclei cells pathologies were revealed (Goryachkina et al., 2017). However, there were separate cell lines, in which the cultures genetic stability was preserved for up to 7 years. According to microsatellite analysis, embryogenic lines were characterized by weak allelomorphic variability (Tretyakova et al., 2017). In general, nonetheless, the capacity of somatic embryos from long-term maintained lines for maturation and germination decreased sooner or later (Tretyakova et al., 2016).

Embryogenic masses long-term cultivation, leading to the formation of somatic embryos and plants, can lead to rare phenotypic anomalies in Picea glauca and P. mariana (Isabel et al., 1996; Tremblay et al., 1999) or to genetic instability in Pinus sylvestris and P. pinaster (Burg et al., 2007; Marum et al., 2009). The changes in the relative content of mitochondrial DNA were observed in embryogenic tissues in Larix leptolepsis, L. decidua, and their reciprocal hybrids (DeVerno et al., 1994). Thus, despite the relatively high stability of embryogenic cultures of representatives of the genus Larix, the quality and quantity of somatic embryos changes over time. In this respect, long-term maintenance of these cultures with the method of regular subcultivation does not make sense.

## Embryogenic lines cryopreservation

Aside from the main issue, which is the decrease or loss of the embryogenic culture potential against the background of possible genetic changes in the course of the long-term maintenance in vitro, it is also worth considering high labor costs if regular subcultivation is necessary; there is also an increasing risk of material loss caused by the pollution, human errors or technical failures.

The embryogenic cultures periodic re-initiation can be the solution to these problems. At the same time, this rather time-consuming and expensive procedure cannot be used for the species, including conifers, for which the most suitable explants for the embryogenic callus induction are available only during limited time of year (Ozudogru, Lambardi, 2016).

Another approach to overcome the forenamed difficulties is to use the technique decreasing the growth rate and increasing the subculture intervals by the way of incubation at a low temperature (4–5 °C) and low light intensity (for example, 10 μmol/m^2^s), change of the conservation medium osmotic potential, reduction of the inorganic nutrients intake, addition of growth retardants to the culture medium (Hassan, 2017). Preservation within the minimal growth conditions is a very simple method to keep the culture in vitro during the periods from 6 to 12 months, but no more than 3 years, depending on the plant species (Ozudogru et al., 2010). Longer crops seasoning under such conditions leads to a sharp drop in the plant regeneration frequency and an increase in the genetic changes number.

For a long-term stable preservation of embryogenic cultures, cryopreservation is an ideal method ensuring their safety and stability (Charest, Klimaszewska, 1995). Embryogenic cultures can be kept in liquid nitrogen at –196 °C or at –150 °C in the nitrogen vapor phase without any time limitations and the juvenility loss (Park et al., 1998). This method provides long-term preservation of various types of tissues and organs, including shoot tips, somatic and zygotic embryos, whole seeds, pollen, anthers and buds (Vendrame, 2018).

There are different types of cryopreservation methods. Traditional methods are based on the freeze-induced dehydration. Among various cryopreservation methods available for embryogenic cultures, the most common approach is slow material cooling. In the last few years this approach has allowed developing some effective protocols for the material preservation without loss or with a slight loss of regenerative capacity for a long time (up to 20 years) for various species such as broadleaf (Citrus ssp., Hevea brasiliensis, Fraxinus excelsior, Quercus suber, Q. robur et al.) and conifers (Abies cephalonica, Picea abies, P. glauca, P. sitchensis, Pinus caribaea, P. nigra, P. patula et al.) (Ozudogru, Lambardi, 2016).

Cryopreservation methods are developed and successfully applied to hybrids of L. × eurolepis and L. × leptoeuropaea (Klimaszewska et al., 1992; Pâques et al., 2013). The use of these methods made possible the achieving of the growth resumption of all tested lines after their thawing. In addition, cryopreservation and its duration (at least 18 years) did not influence noticeably the somatic embryos productivity (Lelu- Walter, Pâques, 2009). Hybrid larch trees cryopreserved lines have been routinely used in experiments for many years. However, until now there is no data on the cryopreservation methods use for long-term preservation of embryogenic lines of the main part of larch species, except for their two hybrid forms. At the same time, the successful experience of the cryopreservation methods use for representatives of other conifer genera creates promising prospects for wider application of this technology for long-term preservation of larch species embryogenic cultures.

## Genetic control of larch trees somatic embryogenesis

At present, in order to explore the molecular mechanisms of the somatic embryogenesis process, a lot of attention is paid to the study of the entire genome profiling on the basis of transcriptomics, proteomics, and metabolomics (Trontin et al., 2016b). Thus, it was shown that during Larix species cell cultures somatic embryogenesis in vitro changes in the mitochondrial genome organization and the relative representation of some genomic regions occur (DeVerno et al., 1994). 454 libraries containing cDNA sequences were created under the study of various stages of somatic embryogenesis of L. leptolepis using the method of RNA sequencing (Zhang Y. et al., 2012). It’s shown that 25773 identified transcripts are connected with 160 biochemical pathways of primary and secondary metabolism. 78 % of genes connected with embryogenesis were completely homologous to those of Arabidopsis thaliana. The genes of the transcription factors LaMYB33 and LaSCL6 are important for the preservation of competence and maintenance of the state of embryogenicity in L. leptolepis as a part of the gene expression regulation epigenetic complex (Li S. et al., 2013; Li W. et al., 2014). During early embryogenesis, the genes LdLEC1 and LdWOX2 (L. decidua) (Rupps et al., 2016), LaSERK1 (L. leptolepis) (Li L. et al., 2013) play an important role. LaNFYA1, LaNFYA2, LaNFYA3, and LaNFYA4 play an important role during early stages of determination and at the beginning of somatic embryo maturation (L. leptolepis) (Zhang L. et al., 2014). At the precotyledonary stage of somatic embryo development, the expression of antioxidant defense genes (SOD, CAT, and APX (L. leptolepis)) is required (Zhang S. et al., 2010a). At the stages of initiation and late maturation of somatic embryos, the expression of genes connected with the auxins synthesis or transport increases: LaHDZ31, 32, 33 and 34 (L. leptolepis) (Li S. et al., 2013; Li Z. et al., 2017b), LaNIT (L. leptolepis) (Li Z. et al., 2018). LmAP2L1 (L. × leptoeuropaea) (Guillaumot et al., 2008), LkBBM (L. leptolepis × L. olgensis) (Li K. et al., 2014), LdBBM and LdSERK (L. decidua) (Rupps et al., 2016) are the most active at the stage of somatic embryo germination. Herewith, the LmAP2L2 gene of the transcription factor is constitutively expressed at all the embryogenesis stages (L. × leptoeuropaea) (Guillaumot et al., 2008).

The highly-productive sequencing strategy was used to identify miRNAs involved in the corresponding target genes regulation at certain stages of somatic embryogenesis in L. leptolepis (Zhang J. et al., 2012). More than 100 target genes have been identified for 60 miRNAs. Differential expression of different miRNAs (miR156, miR159, miR160, miR162, miR165, miR166, miR167, miR168, miR169, miR171, miR172, miR397, miR398) was found in embryogenic and non-embryogenic L. leptolepis cultures (Zhang J. et al., 2012; Zhang L. et al., 2014; Li S. et al., 2013; Li W. et al., 2013, 2014). In conifers, miRNAs regulate the activity of most genes of transcription factors, including genes involved in the embryogenesis process. The transcription factor MYB (LaMYB33) was identified as a target gene for miR159 (Li W. et al., 2013). In its turn, LaHDZ31, LaHDZ32, LaHDZ33, and LaHDZ34 are regulated by miR165/166 (Li Z. et al., 2016). MiR169 targets are LaNFYA1, LaNFYA2, LaNFYA3 and LaNFYA4 (Zhang L. et al., 2014), and the homologue Larix SCARECROW-LIKE 6 (LaSCL6 ) is a target for miR171 (Zang et al., 2019). These genes post-transcriptional regulation with miRNA can be involved in maintaining the developmental potential, as described above, at various stages of somatic embryogenesis in representatives of the genus Larix. The genes identified in representatives of the genus Larix, playing an important role in somatic embryogenesis at different stages, are summarized in Table 2.

**Table 2.**
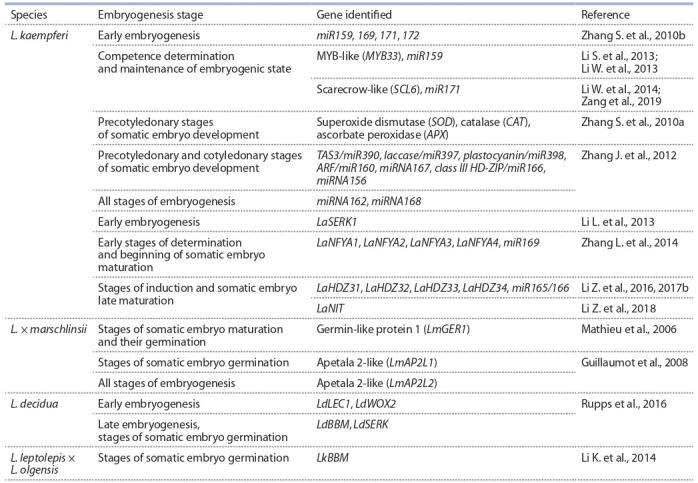
Genes involved in somatic embryogenesis in representatives of the genus Larix

Considering the future prospects of genomic studies of Larix species cell cultures somatic embryogenesis, it is possible to note the fundamental importance of clarifying such important theoretical and applicative issues as (1) structural and functional changes in the mitochondrial genome of larches occurring in culture in vitro, and the possibility of their reversion; and (2) genetic regulation of the interaction of the nucleus, mitochondria and chloroplasts genomes during somatic embryogenesis. It is obvious that the success of the development of this technology for cell culture in vitro will largely depend on the resolution of the issues mentioned above.

Work to determine changes in the proteome and composition of fatty acids at different development stages of embryogenic masses and somatic embryos of a number of larch species was carried out. Comparative proteomic analysis of embryogenic and non-embryogenic L. principis-rupprechtii calli revealed 503 proteins, 71 of which were differentially regulated (Zhao et al., 2015b). In addition, proteins were analyzed at three stages of somatic embryo development of the same larch species: stages of proembryogenic mass, globular and cotyledonary embryos. 96 proteins differentially expressed at different stages of development were identified. Functional analysis showed that the content of proteins involved in the primary metabolism, phosphorylation, and maintenance of the cellular redox potential, increases during the development of somatic embryos. The study of the general profile of L. × eurolepis proteins showed significant differences in their content at certain stages of somatic embryo maturation (Teyssier et al., 2014). It was found that the 147 proteins found in the work are mainly involved in the primary metabolism and stabilization of the resulting metabolites. Thus, storage proteins identified as legumin- and vicilin-like appeared at the precotyledonary stage of development.

When studying the fatty acid composition of the lipids of embryogenic and non-embryogenic L. sibirica calli, a high content of oleic acid in total lipids of the embryogenic cell culture was found against the background of a lower content of linoleic acid compared to the non-embryogenic callus (Makarenko et al., 2016). The review proposes to use the concentration of these fatty acids as a marker of embryogenic potential in the selection of promising cell lines of Siberian larch during early embryogenesis. Significant differences in the composition and content of neutral lipids in tissues of embryogenic and non-embryogenic L. sibirica cell lines were also revealed (Semenova et al., 2020). Glycerides were found to be dominant lipids of two types of lines. Triglycerides and 1,2-diglycerides accumulated more actively in embryogenic cell lines, while the content of sterol esters in these lines was reduced.

Nevertheless, despite significant advances in understanding the molecular genetic mechanisms underlying somatic embryogenesis in conifers, in particular representatives of the genus Larix, there is currently an urgent need to expand complex research in this field of study in order to get new knowledge necessary for the development of methods and approaches to obtaining plant material in vitro and using it in reforestation and afforestation programs.

## Use of the method of somatic embryogenesis in representatives of the genus Larix in reforestation breeding programs

The method of individual conifer species microclonal propagation by somatic embryogenesis has already begun to be widely used in various countries of the world, primarily in France, Canada, Germany, Great Britain, Ireland, Scandinavian countries, China, in plantation forestry and in the implementation of MVF (Multi Variety Forest) programs (Park et al., 2016). MVF is defined as the use of a range of genetically tested woody species in a production forest seed orchard (Weng et al., 2011). Back in the mid-90s of the last century, INRA (National Institute for Agricultural Research) in France organized studies on somatic embryogenesis in hybrid larch species (Pâques et al., 2013). This led to the development of an improved procedure leading to the routine production of regenerant plants from somatic embryos. The new protocol was applied for the hybrid L. × leptoeuropaea cultivar REVE-VERT propagation (Lelu-Walter, Pâques, 2009). In the same years, a program of breeding hybrid L. × eurolepis was launched at the state enterprise “Staatsbetrieb Sachsenforst” (Germany), based on the achievements in the field of combining the method of clonal propagation and the original plant material having an excellent genetic background (controlled crosses) (Kraft, Kadolsky, 2018). Breeding programs, including biotechnological approaches of somatic embryogenesis and genetic engineering, were launched in China and aimed at improving the existing gene pool of the local species L. principis-rupprechtii (Zhao et al., 2015a). Genetic engineering methods in conjunction with somatic embryogenesis of larch trees were successfully tested in laboratory conditions a long time ago. At the same time, both agrobacterial and bioballistic transformation were used to introduce genes into cells of embryogenic culture and directly somatic embryos of L. laricina, L. leptolepis, L. × eurolepis, L. principis-rupprechtii (Klimaszewska et al., 1997; Levée et al., 1997; Qi et al., 2000; Li Z. et al., 2016). In the next while, for the genetic improvement of conifers, it is planned to use the rapidly developing and promising genomic editing technologies based on the CRISPR-Cas system along with traditional methods of transformation. These technologies have already been successfully applied to broad-leaved tree species, such as grapefruit (Cītrus paradīsi), orange (Cītrus × sinēnsis), apple (Malus domestica, M. prunifolia × M. pumila), poplar (Populus tomentosa, P. tremula × alba, P. tremula × tremuloides), and others (Sarmast, 2016; Chang et al., 2018).

The technology of genomic selection has been successfully used in recent decades in order to increase the efficiency of tree selection, along with the methods of somatic embryogenesis and cryopreservation (Park, 2002). This technology, based on a set of mapping of quantitative traits loci, makes possible the prediction of the phenotype of an individual (Goddard, Hayes, 2007). Thus, genomic selection makes it possible to identify elite genotypes at a very early stage of development without phenotyping through field testing and, thereby, to reduce significantly the duration of variety testing within the framework of afforestation programs (Park et al., 2016).

In our country, thanks to intensive studies of the conifers somatic embryogenesis, including Siberian larch species, conducted for more than 10 years by the staff of the Sukachev Institute of Forest SB RAS (Krasnoyarsk) under the leadership of I.N. Tretyakova, significant achievements have been made in this area, starting from the conditions for the embryogenic culture induction up to the carrying out of field testing of plants grown from somatic embryos – regenerants (Tretyakova et al., 2018). The results obtained along with the use of progressive methods of cryopreservation, creation and selection of elite genotypes based on genetic engineering and genomic selection can significantly increase the effectiveness of traditional reforestation breeding programs carried out in Russia. The international project “Larch” with the participation of Sweden, Norway, Finland, Iceland, Canada, China, Japan, and the United States can be considered as the most ambitious of these projects (Abaimov et al., 2002; Martinsson, 2002). The aim of this project, launched in 1992, is to create a collection of seeds of four larch species from Russia (Larix sukaczewii, L. sibirica, L. gmelini and L. cajanderi), study the genetics of these species, carry on research on the breeding and modification of populations created on plantations in different parts of the Northern hemisphere, and select promising forms and populations.

## Conclusion

Somatic embryogenesis is increasingly considered as the most promising method of clonal propagation of conifers (in particular, larches) since it has a number of advantages over traditional afforestation technologies. For more than 30 years since the first successful somatic embryogenesis in representatives of the genus Larix, considerable experience has been accumulated in this research area. The conditions of induction, maintenance of embryogenic cultures, maturation and germination of somatic embryos, regeneration of full-fledged plants and their growth in field conditions have been studied. Based on cryopreservation methods, protocols for maintaining embryogenic cultures in a functional juvenile state for long periods of time have been developed. Research is being actively carried out to elucidate the molecular genetic mechanisms underlying the implementation of individual stages of somatic embryo development.

Despite the existing successes, today it is required to intensify comprehensive research in the field of somatic embryogenesis of representatives of the genus Larix. In the nearest future, the research will provide the creation of scientific foundations for the development of new approaches and methods for obtaining high-quality plant material in vitro and its use in extensive industrial reforestation and afforestation programs. Hopefully, integrating efforts and closer cooperation of individual research teams successfully working in the Russian Federation on the problem of somatic embryogenesis of species of the genus Larix and other conifer species, as well as the already begun use of an integrated approach based on the use of genomics, transcriptomics, proteomics, metabolomics, cryopreservation, and genomic selection in these studies will accelerate the resolution of this planetary biological problem.

## Conflict of interest

The authors declare no conflict of interest.
